# Budget Impact Analysis: Digital Workflow Significantly Reduces Costs of Implant Supported Overdentures (IODs)

**DOI:** 10.1111/cid.13413

**Published:** 2024-11-13

**Authors:** Thomas Van de Winkel, Frans Delfos, Bart van Oirschot, Thomas Maal, Eddy Adang, Gert Meijer

**Affiliations:** ^1^ Department of Oral Maxillofacial Surgery Radboud University Medical Center Nijmegen The Netherlands; ^2^ Dental Laboratory of the Department of Dentistry Radboud University Medical Center Nijmegen The Netherlands; ^3^ Department of Dentistry‐Regenerative Biomaterials Radboud University Medical Center Nijmegen The Netherlands; ^4^ Department of Health Evidence, Health Evidence Member‐Associate Professor Health Economics Radboud University Medical Center Nijmegen The Netherlands

**Keywords:** budget impact analysis (BIA), CAD/CAM, cost consequence analysis (CCA), digital workflow, edentulous, implants, overdenture

## Abstract

**Background:**

For edentulism, an implant supported removable complete overdenture (IOD) is an attractive solution to restore patients' chewing capacity, aesthetics, and self‐esteem, however, treatment is expensive and time consuming.

**Purpose/Aim:**

To estimate the decline in costs for digitally designed and CAD/CAM fabricated IODs (3D‐IODs) compared to conventionally fabricated IODs (C‐IODs) at comparable general health related quality of life (GHRQoL).

**Materials and Method:**

A randomized crossover study enrolled 36 fully edentulous patients, in whom six maxillary implants were placed together with two mandibular implants, if not already present.

At the start of the study, a set of C‐IODs and 3D‐IODs was fabricated for each patient. All patients wore each IOD‐type for 1 year: first the 3D‐IOD and the second year a C‐IOD, or vice versa. At all three‐time points patients general QoL was assessed using the EQ‐5D‐5L questionnaire as well as the SF‐36 from which the SF‐6D was obtained, to research the anticipation of no significant difference.

To enable cost consequence analysis (CCA), both costs made within healthcare and patient costs were assessed. Subsequently, a budget impact analysis (BIA) was performed to demonstrate the potential savings.

**Results:**

No differences in general GHRQoL were seen between C‐IOD (*M* = 0.840, SD = 0.177) and 3D‐IOD (*M* = 0.837, SD = 0.156) (paired *t*‐test (*N* = 31): *p* = 0.880).

With respect to the total costs for a complete IOD, however, the digital approach showed a reduction in initial total costs of 14.2% (€4700.33 vs. €4030.61: *p* < 0,001), in treatment time of 41.1% (309 vs. 182 min: *p* < 0.001), and in number of treatment sessions of 47.1% (5.68 vs. 3.0: *p* < 0.001). For repairs for an IOD in both the upper and lower jaw, the C‐IOD and 3D‐IOD scored similar for treatment time as well as additional costs.

**Conclusion:**

Implementing a 3D workflow in the production of IOD's supplies patients with a high‐quality 3D‐IOD at lower costs.

**Trial Registration:** NL‐OMON44248 https://onderzoekmetmensen.nl/en/trial/44248

## 1 Introduction

### 1.1 Edentulism

Edentulism induces functional and esthetic burden [1]. In contrast to the overall trend towards a decrease in edentulism, the group in greatest need of complete oral rehabilitation is the rapidly growing aging population [2]. The World Health Organization (WHO) corroborated that 22% of the world's population will be older than 60 years by 2050 [3]. Therefore, need for rehabilitation of edentulous patients is likely to remain relevant for the foreseeable future [4].

### 1.2 Cost Reduction of a Digitally Versus Conventionally Fabricated Implant Supported Removable Overdenture (IOD)

**FIGURE 1:**
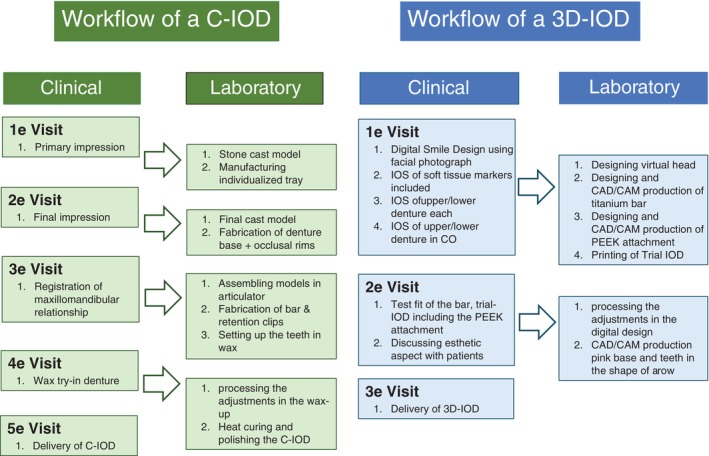
Representation of the fabrication of a C‐IOD (5 visits) versus a 3D‐IOD (3 visits).

Using computer aided design and computer aided manufacturing (CAD/CAM), traditional clinical and laboratory steps can be replaced, making prosthodontic treatments faster and cheaper [5]. The ability to integrate a variety of digital files such as a cone beam computer tomography (CBCT) scan, an intra oral scan (IOS) and a facial image into the same software package enables to digitally design an IOD (3D‐IOD) already after one clinical session (Figure 1). During the second clinical visit, a trial‐IOD can be extensively tested and, if necessary, adjusted. The final 3D‐IOD is then delivered in the third session. For the conventional workflow, however, to fabricate an IOD (C‐IOD) five sessions are needed (Figure 1) [6]. As an advantage, CAD/CAM skips polluting laboratory steps such as using polymers for mixing PMMA before heat‐pressing. Furthermore, intraoral scanning eliminates the need for conventional impression techniques, by which patient comfort is significantly increased [7].

### 1.3 Economics in Relation to Patient Reported Outcome Measures (PROMs)

Health economic analyzes provide information to control costs, not only private costs, but also those related to the public sector in the case of subsidized dental care [8].

For measuring oral health related quality of life (OHRQoL), the Oral Health Impact Profile (OHIP‐20) is the most comprehensive questionnaire and widely used [9]. However, in order to be a comprehensive tool for economic evaluations, a questionnaire should provide preference‐based measures (index scores, or “utilities”), meaning that the scores (ranging from 0 to 1) represent the value of a health status based on societal preferences [10, 11]. Unfortunately, the OHIP questionnaires are not preference‐based [9]. Only a few questionnaires, which focus exclusively on assessing general health‐related quality of life (GHRQoL), meet this requirement [10, 11, 12].

One of the key preference based measures is the EuroQol 5D‐5L (EQ‐5D‐5L) [12]. Consisting of only five questions however, it is likely to be less sensitive [12]. The SF‐36 is a more in‐depth health survey with 36 items for assessing GHRQoL [13]. To enable economic evaluation, a preference‐based index can be derived from the SF‐36, referred to as the SF‐6D [12].

The aim was to calculate the difference in costs between C‐IODs and 3D‐IODs and at the same time to demonstrate that both IOD‐types lead to a comparable GHRQoL. For interventions that are equally or more effective and cost less, identifying the preferred intervention is rather straightforward, and no incremental cost‐effectiveness ratio (ICER) is needed [14]. In those cases a cost consequence analysis (CCA) can be performed.

## 2 Material and Methods

The present study was conducted in accordance with the Declaration of Helsinki and approved by the Ethics Committee of Arnhem/Nijmegen, 2017–3671, 12 December 2017 (Dossier number: 2017–3671 NL‐number: NL63073.091.17).

The article was composed according the “Consolidated Health Economic Evaluation Reporting Standards 2022” [15]. The raw data are archived in the “Data Archiving and Networked Services” (DANS) of the Royal Netherlands Academy of Arts and Sciences (KNAW) under the Persistent identifier 10.17026/dans‐25s‐6cdk and hence are accessible for the public.

### 2.1 Study Population

In case of edentulism, Dutch insurance companies reimburse solely IODs, provided that all other prosthetic options have failed, and at the same time extreme resorption of the alveolar ridge can be confirmed radiographically.

Inclusion criteria comprised edentulism in combination with (a) severe retention problems of the upper conventional denture (CD) caused by extreme maxillary bone resorption, requiring bone augmentation prior to implant placement, (b) approved permission from the insurance company to perform bone reconstruction, implant installation, and fabrication of the IOD, (c) an IOD already present or planned in the edentulous lower jaw, (d) willingness to participate in the study, and (e) having signed an informed consent. Exclusion criteria were (a) clinical signs of severe oral dysfunction, (b) systemic diseases/conditions that compromise successful implant therapy, (c) smoking (d) non‐compliance with the study protocol.

### 2.2 Centers

Two centers were involved: Radboud university medical center (Radboudumc) in Nijmegen, the Netherlands and the Amphia general hospital in Breda, the Netherlands.

### 2.3 Implants

In all augmented upper jaws, bone volume was sufficient at the time of implantation. In Nijmegen, in total 4–6 Nobel Parallel Conical Connection implants (Nobel Biocare, Kloten, Switzerland) were installed according to the NobelGuide procedure (Nobel Biocare, Kloten, Switzerland), which means that the implants were placed without a flap. In the lower jaw, if indicated, two implants of the same brand were installed using an open procedure, by first preparing a subperiosteal flap.

In Breda, both in the lower and upper jaw, patients received Straumann Tissue Level Implants (Standard Plus) with a diameter of 4.1 mm (Institute Straumann AG, Basel, Switzerland). Implant placement was executed in an open procedure.

Immediately after implant installation, onto all implants healing abutments were positioned. 2 weeks later the patients were allowed to wear their CD again, which first was modified for this purpose.

### 2.4 Trial Design

In this randomized cross‐over study, patients were asked to wear the C‐IOD and the 3D‐IOD‐type alternately for 1 year each. The Institute for Medical Technology Assessment Productivity Cost Questionnaire (iMTA PCQ) questionnaire, the EuroQoL‐5D‐5L (EQ‐5D‐5L) health questionnaire and the 36‐Item Short Form Health Survey (SF‐36) were accessed prior to the study and after wearing each denture for 1 year. The OHIP‐20 was the primary outcome of this research, as described in a previous article, in which a detailed sample size calculation was presented [16]. This resulted in a study population of 36 persons, accounting for a possible dropout of six patients [16].

All patients were randomly assigned; 18 participants to the A‐group, who started with the 3D‐IOD and switched to the C‐IOD after 1 year. Another 18 participants (B‐group) began with the C‐IOD and ended with the 3D‐IOD. Assignation to Group A or B was executed with a 1:1 allocation as per a computer‐generated randomization schedule stratified (male, female) and using permuted blocks of random sizes. To ensure concealment, the block sizes were not disclosed. Participants were randomized using an online randomization tool [16].

Treatment sessions were combined to hide from the patient which techniques were used for which IOD‐type. At the start of the study, both the C‐IOD and the 3D‐IOD were finalized for each patient. Which IOD‐type was placed remained out of sight for both patient and researcher.

### 2.5 EQ‐5D‐5L

The EuroQol 5D‐5L (EQ‐5D‐5L) is a standardized GHRQoL instrument that scores on five general health levels: Mobility, self‐care, daily activities, pain/discomfort, and anxiety/depression [12] From this, a weighted health index can be derived for an individual or population. The five questions about health status are scored on a 3‐point scale (1–3). This can be converted into a health index ranging from 0 to 1. As the population consists of Dutch patients, the scores were converted to a health index using EuroQol's value set for the Netherlands [17, 18].

Finally, the patients had to range their general health situation on a scale from 0 to 100 (VAS‐score). They were asked to complete the questionnaire before the start of treatment, after 12 months and at the end of the study after 24 months.

### 2.6 SF‐36 and SF‐6D

The SF‐36 is a health survey for assessing the experienced level of health or GHRQoL. The instrument consists of separate levels for physical functioning, bodily pain, role limitations due to physical health problems, role limitations due to personal or emotional problems, emotional well‐being, social functioning, energy/fatigue, and general health perception [13].

The SF‐36 is one of the most widely used measures for health related quality of life [10]. To perform an economic evaluation based on the SF‐36, the SF‐6D health state classification needs to be derived from the SF‐36 [10, 11].

### 2.7 Costs

It is important to emphasize that only the costs of the IOD itself were calculated, so the implant treatment, including the implants themselves, were not included as these are the same for both IOD‐types. Bottom‐up micro‐costing, a valuation technique which starts with a detailed identification and measurement of all the inputs consumed in a health care intervention and all of its sequelae [19], is generally regarded as the most detailed method, as all relevant units of care are identified, and each individual unit of care is valued for individual patients [20, 21].

Costs for manufacturing and repair of both a C‐IOD and a 3D‐IOD consisted of the dentist's fee and the dental laboratory costs. Fixed prices applied for both, which are determined annually by the Dutch Healthcare Authority (NZa). Since the dentures were fabricated in 2018 and 2019, the rates for dental care for these years were used. To calculate the laboratory costs, the actual invoices of the dental laboratories were used. In addition, patient travel costs and productivity losses for both paid and unpaid work were assessed in monetary terms.

### 2.8 Institute for Medical Technology Assessment Productivity Cost Questionnaire ( *i*MTA PCQ)

The *i*MTA PCQ is the leading Dutch tool to measure and assess productivity losses in the Netherlands [22]. It is a short and concise instrument suitable for quantifying presenteeism (“being present at work when sick”) and absenteeism (“taking sick leave”) and relates to both loss of productivity in case of “paid work,” as well as “unpaid work” [23, 24].

To objectivate the productivity losses of both paid and unpaid work, the Dutch National Health Care Institute's Guideline for economic evaluations in healthcare was used, which determined the average labor costs per hour for paid work at €34.75 (€36.52 after inflation correction to 2018, the year in which the first IOD's were manufactured) and the replacement costs for unpaid work at €14.00 (€14.71 after inflation correction) [25].

The iPCQ was asked to be completed at baseline and after 12 and 24 months (12 months after switching).

### 2.9 Budget Impact Analysis (BIA)

Purpose of the BIA was to assess the financial impact of implementing 3D‐IODs to replace C‐IODs in the Dutch health care system in the short‐to‐medium term from the budget holder's perspective [26]. For this goal, the framework was used as presented in the Dutch guideline for economic evaluations [25]. Impact of the new treatment mix on prevalence and incidence was investigated by use of epidemiological data in the Dutch context.

In the BIA, market dynamics were considered, such as estimates of uptake of the treatment mix above. In total three scenarios were presented: Current care, immediate inclusion of 100% 3D‐IODs versus gradual implementation in steps of 25% uptake. Furthermore, BIA base‐case perspectives, society, health insurance/third party payer, and health care were considered. Both annual resource use (in terms of volumes consumed) and cost (volumes multiplied by prices) were presented.

To inventory the amount of care, meaning the number of implant‐related treatments that were reimbursed by Dutch health insurers in the years 2013–2021, the associated codes were requested from the National Health Care Institute (ZIN). To perform the BIA, the latest published numbers of 2021 of treatment codes were used. Prices were extracted from the list “Maximum reimbursements for material and technique costs for implantology and suprastructure, January 2021 up to and including 31 December 2021” [27].

To inventory the relationship between the use of solitary abutments and a bar construction, an estimation was made based on consultations with oral maxillofacial surgeons, dental technicians, advising dentists for assurance compagnies and Dutch professors in oral implantology.

### 2.10 Statistics

For the statistical analyses the 27th version of SPSS was used (IBM Corp. Released 2020. IBM SPSS Statistics for Windows, Version 27.0. Armonk, NY: IBM Corp). Regarding costs as well as treatment time, treatment sessions, travel time, and patient satisfaction, differences between the C‐IOD and 3D‐IOD were calculated using a paired sample *t*‐test. When subgroups with less than 22 samples were analyzed, the test was bootstrapped by 1000. For the EQ‐5D, an analysis of variance (ANOVA) for crossover study was conducted to determine if the GHRQoL was different for the C‐IOD versus 3D‐IOD group. Only descriptives were performed for the *i*MTA PCQ since the number of working hours in this population turned out to be too low.

## 3 Results

### 3.1 Patients

A total of 29 patients were treated in the Radboudumc, Nijmegen, The Netherlands and seven at the Amphia hospital, Breda, The Netherlands. Of these participants, 32 completed the two‐year follow‐up, four patients dropped out of the study; two patients died, one was hindered from traveling due to vision difficulties, and the fourth moved abroad. In total 16 men and 16 women completed the study; they started at a mean age of 62.8 years (SD: 6) ranging from 51 to 75 years (Table 1).

**TABLE 1:** Demographics at baseline.

	*N*	Minimum	Maximum	Mean	Std. dev.
Male	16	53	75	63.63	6.84
Female	16	51	71	62.00	6.49
Group A	15	59	75	65.40	4.84
Group B	17	51	71	60.53	7.24
Total	32	51	75	62.81	6.61

*Note:* Group A = started with 3D‐IOD, Group B = started with C‐IOD.

### 3.2 Implants

Of the installed 181 maxillary implants, four failed during osteointegration, two of which occurred in one patient. Another implant was lost during the first year. All were Nobel Parallel Conical Connection implants which equates to a failure rate of 2.8% after 2 years of function. In the lower jaw, 68 mandibular implants were present (30 patients with two implants, 2 patients with four implants), none of which failed.

### 3.3 Prosthetics

All 32 patients received a 3D‐IOD in their upper jaw, 22 also in their lower jaw. For 10 patients, it was not possible to make a mandibular 3D‐IOD, because implants of relatively unknown brands were present, which were not recognized by the TRIOS Design Studio program (3Shape).

For the mandibular C‐IOD, in Nijmegen, a milled titanium bar with distal extensions (Atlantis, Charlotte, North Carolina, USA) together with a Dolder matrix (Cendres Metaux, Biel, Switzerland) was used. For the maxillary C‐IOD, preference was given to the prefabricated Locator system (ZEST Anchors, Carlsbad, USA).

In Breda, for the mandibular C‐IOD solely a milled titanium bar with distal extensions (Atlantis, Charlotte, USA) was placed as suprastructure. A similar bar was also used as attachment for the maxillary C‐IOD.

All patients were able to wear both IOD‐types till the end of the study, achieving a 100% survival rate (SR) from a prosthetic point of view. Related to its design and attachment, each IOD‐type had its own prosthetic complications as described in Table 2.

**TABLE 2:** Types of prosthetic complications, as scored for the C‐IOD and 3D‐IOD, in both the upper and lower jaw.

Complications	Upper C‐IOD	Upper 3D‐IOD	Lower C‐IOD	Lower 3D‐IOD
Pressure ulcer	18	6	26	6
Rebasing	3	—	—	—
Optimizing PEEK retention	—	1	—	5
Loose matrix (housing)	11	—	—	—
Changing nylon rings Locator	9	—	—	—
Changing Locators	2	—	—	—
Loose screws (bar)	—	1	1	11
Fracture of screw	—	—	—	7
Loose ball or VKS attachment	—	—	14	—
Clip activation	—	—	5	—
Tear/Fracture of denture base	1	1	2	2
Fracture of the bar	—	—	1	—
Correction of occlusion	3	6	—	8
Changing position upper teeth	—	1	—	—
Total number of complications	**47**	**16**	**49**	**39**

*Note:* The different colors was to distinguish between both upper and lower jaw and C‐IOD and 3D‐IOD.

### 3.4 Cost Consequence Analysis (CCA)

#### 3.4.1 Costs Made Within Healthcare—Production of the IOD

With respect to both the upper and lower IOD, the digital approach showed a reduction in total costs of 14.2% (€4700.33 vs. €4030.61: *p* < 0.001), in treatment time of 41.1% (308.64 vs. 182.05 min: *p* < 0.001), and in number of treatment sessions of 47.1% (5.68 vs. 3.0: *p* < 0.001) (Tables 3 and 4).

**TABLE 3:** C‐IOD versus 3D‐IOD: Mean costs for both initial production and repair.

Costs								
	Details of IOD manufacturing						Details of IOD reparation	
	Total costs	Std. deviation	Lab costs	Std. deviation	Honorarium	Std. deviation	Lab costs	Std. deviation
(*N* = 22)	Upper jaw: Locators C‐IOD vs Bar 3D‐IOD + Lower jaw: Bar C‐IOD versus Bar 3D‐IOD							
**C‐IOD**	€4.700.33	€668.15	€3.535.63	€584.41	€1.164.70	€143.38	€49.56	€168.26
**3D‐IOD**	€4.030.61	€212.16	€2.947.95	€131.35	€1.082.66	€98.06	€26.58	€53.00
*p*‐value	< 0.001*		< 0.001*		0.062		0.551	
Std. deviation	€792.24		€625.59		€194.81		€177.07	
95% conf. interval	€318.46–€1020.98		€310.31–€865.05		€−4.33 to €168.41		€‐55.81–€101.77	
(*N* = 27)	Upper jaw: Locators C‐IOD vs Bar 3D‐IOD							
**C‐IOD**	€2.410.96	€209.79	€1.764.08	€253.14	€646.87	€168.71	€1.38	€7.16
**3D‐IOD**	€2.383.37	€72.50	€1.759.63	€84.78	€623.74	€84.44	€1.66	€5.96
*p*‐value	0.532		0.924		0.244		0.881	
Std. deviation	€226.07		€239.91		€100.72		€9.57	
95% conf. interval	€−61.85 to € 117.01		€−90.45 to € 99.36		€−16.71 to €62.97		€−4.063 to €3.508	
(*N* = 5)	Upper jaw: Bar C‐IOD vs Bar 3D‐IOD							
**C‐IOD**	€3.659.70	480.44	€2.846.60	490.31	€813.10	144.20	€19.96	€44.63
**3D‐IOD**	€2.239.41	237.29	€1.644.30	140.76	€595.11	127.25	€0.00	€0.00
*p*‐value	< 0.010* ^a^		0.089^b^		0.052^c^		0.083^d^	
Std. deviation	€276.79		€349.63		€160.12		€44.63	
95% conf. interval	€1221.05–€1659.64^a^		€938.90–€1462.50^b^		€19.18—€416.81^c^		€19.69–€59.88^d^	
(*N* = 22)	Lower jaw: Bar C‐IOD vs Bar 3D‐IOD							
**C‐IOD**	€2.106.35	€208.00	€1.579.58	€192.37	€526.78	€49.83	€43.34	€167.76
**3D‐IOD**	€1.695.03	€126.38	€1.200.73	€76.11	€494.30	€71.34	€24.55	€52.44
*p*‐value	< 0.001*		< 0.001*		0.065		0.630	
Std. deviation	€237.84		€175.84		€78.15		€180.38	
95% conf. interval	€305.87–€516.78		€300.89–€456.81		€−2.17 to €67.11		€−61.19 to €98.76	

*Note:* Bootstrap: a = bootstrapped based on 997 samples, b = based on 986 samples, c = based on 999 samples, d = based on 669 samples. The different colors was to distinguish between C‐IOD and 3D‐IOD and between manufacturing on the one hand and reparation on the other hand.

* Significant (*p* < 0.05) difference in paired *t*‐test.

**TABLE 4:** C‐IOD versus 3D‐IOD: Sessions and treatment time for both manufacturing and repair.

Treatment								
	Details of IOD manufacturing				Details of IOD reparation			
	Sessions (*n*)	Std. deviation	Time (min)	Std. deviation	Sessions (*n*)	Std. deviation	Time (min)	Std. deviation
(*N* = 22)	Upper jaw: Locators C‐IOD vs Bar 3D‐IOD + Lower jaw: Bar C‐IOD versus Bar 3D‐IOD							
**C‐IOD**	5.68	0.478	308.64	51.644	4.59	3.53	125.91	108.93
**3D‐IOD**	3.00	0.000	182.05	9.839	3.45	2.46	102.27	84.21
*p*‐value	< 0.001*		< 0.001*		0.037		0.103	
Std. deviation	0.48		53.26		2.40		65.09	
95% conf. interval	2.470–2.893		102.98–150.21		0.74–2.20		−5.22–52.49	
(*N* = 27)	Upper jaw: Locators C‐IOD versus Bar 3D‐IOD							
**C‐IOD**					1.63	1.52	47.4	47.38
**3D‐IOD**					1.66	1.49	47.8	47.03
*p*‐value					0.327		0.327	
Std. deviation					0.19		1.92	
95% conf. interval					−0.113–0.039		−1.13–0.39	
(*N* = 5)	Upper jaw: Bar C‐IOD versus Bar 3D‐IOD							
**C‐IOD**					2.0	3.08	31.0	44.22
**3D‐IOD**					0.2	0.44	14.0	21.91
*p*‐value					0.309^a^		0.463^b^	
Std. deviation					3.03		54.04	
95% conf. interval					0.40–4.60^a^		−22.00–57.00^b^	
(*N* = 22)	Lower jaw: Bar C‐IOD versus Bar 3D‐IOD							
**C‐IOD**					2.55	2.39	72.0	76.65
**3D‐IOD**					2.32	1.89	68.2	60.98
*p*‐value					0.646		0.731	
Std. deviation					2.29		51.96	
95% conf. interval					−0.787–1.24		−19.17–26.90	

*Note:* Bootstrap: a = based on 934 samples, b = based on 993 samples. The different colors was to distinguish between C‐IOD and 3D‐IOD and between manufacturing on the one hand and reparation on the other hand.

* Significant (*p* < 0.05) difference in paired *t*‐test.

The total costs for a maxillary C‐IOD on Locators and a maxillary 3D‐IOD on a bar (*n* = 27) were comparable, namely ± €2400. This also applied to the laboratorial costs (± €1760) for both IOD‐types (Table 3).

As compared to the maxillary bar‐retained C‐IOD, for the bar‐retained 3D‐IOD total costs were significantly less (39%; €3659.70 vs. €2239.41: *p* < 0.01), for honorarium 26.8% (€813.10 vs. €595.11; *p* = 0.052), and for laboratorial costs even 42.2% (€2846.60 vs. €1644.30; *p* = 0.089) (Table 3).

Regarding the lower jaw, when focussing on the patients (*N* = 22) who wore both the mandibular C‐IOD and the mandibular 3D‐IOD, total costs were 20% less (€2106.35 vs. €1695.03: *p* < 0.001) and laboratorial costs even 24% (€1579.58 vs. €1200.73: *p* < 0.001; Table 3).

#### 3.4.2 Costs Made Within Healthcare—Maintenance and Repair

For an IOD in both the upper and lower jaw together, the C‐IOD and 3D‐IOD scored comparable for time (125.91 vs. 102.27 min; *p* = 0.103) (Table 4), as well as for additional lab costs (€49.56 vs. €26.58; *p* = 0.551) (Table 3), but the IOD scored slightly better in number of reparation sessions (4.59 vs. 3.45; *p* = 0.037) (Table 4).

With respect to a bar‐retained C‐IOD versus a bar‐retained 3D‐IOD in the lower jaw, no statistical difference was found for the number of reparation sessions (*p* = 0.646), reparation time (*p* = 0.731), or “extra costs” (*p* = 0.630; Tables 3 and 4).

Also, for the maxillary C‐IOD on locators versus the bar‐retained 3D‐IOD (Table 4), no statistical difference was found for the number of reparation sessions (*p* = 0.327), or time (*p* = 0.327), or “extra costs” (*p* = 0.881). The same applied to the bar‐retained C‐IOD versus the bar‐retained 3D‐IOD in the upper jaw (Tables 3 and 4).

#### 3.4.3 Costs on Patient Level Made by Patients/Family: Real Travel Costs, Out‐Of‐Pocket Expenses, and Time Costs

Each trip to the clinic costed an average of €13.90 with an average travel time (there and back) of 70 min. Creating a full set of C‐IODs took an average of 5.68 sessions, followed by 4.59 repair sessions. For each patient, this meant 10.3 trips at €13.90 per trip (in total €142.75) with a travel time of 719 min. Together with a total treatment time of 308.64 min, and total repair time of 125.91 min, this resulted in a production loss of a total of 1153.45 min (19.2 h) per patient.

For a full set of 3D‐IODs, three clinical sessions were needed, followed by 3.45 repair sessions, implicating an average of 6.45 trips at €13.90 per trip (in total €89.66) with a duration of 451.5 min. As the average total treatment time was 182 min, the average repair time 102 min, and the travel time of 452 min, this meant a production loss of 736 min (12.3 h) per patient.

#### 3.4.4 Costs of Productivity Loss due to IOD Manufacturing

##### 3.4.4.1 Productivity Loss in Case of Paid Work

The *i*MTA‐PCQ analysis showed that during the research period of 2 years, eight (25%) persons received salary for 31 working hours a week while wearing the 3D‐IOD and for 28 working hours a week while wearing the C‐IOD (Table 5).

**TABLE 5:** Outcome iMTA‐PCQ differentiated for the period the C‐IOD and 3D‐IOD were worn.

Outcome iMTA‐PCQ questionnaire	3D‐IOD	C‐IOD
Paid work: number of patients with paid work	8	8
Paid work: percentage of patients with paid work	25%	25%
Paid work: average hours of work per week	31	28
Paid work: average hours of work per day	7.0	7.0
Paid work: patients (*n*) who did work, although having complaints	3	2
Paid work: average worked days (*n*) despite having complaints	9.7	15
Paid work: percentage that patients could work despite discomfort	60%	90%
Unpaid work: patients (*n*) who were limited by complaints	9	7
Unpaid work: days (*n*) on which less work could be done	16.6	26.7
Unpaid work: hours (*n*) of unpaid work that were taken over	1.6	2.8

According to the Dutch National Health Care Institutes guideline on economic evaluations [28] productivity costs are considered at €36.52 per worked hour. Only eight of the patients (25%) has paid jobs, both in the C‐IOD and 3D‐IOD group. The mean productivity loss for making the C‐IOD, reparations included, was 19.2 h. Multiplied by the productivity costs of €36.52 per hour, this amounts to a total €701.18 per worker for the C‐IOD‐group and €447.98 per worker for the 3D‐IOD‐group (12.3 h at €36.52 per hour).

One person was ill for less than 4 weeks while wearing a 3D‐IOD and one other person when wearing a C‐IOD. Since the illness period was shorter than 4 weeks, according to the Dutch guidelines, it was not indicated to apply the friction cost method.

##### 3.4.4.2 Productivity Loss in Case of Complaints

While wearing a 3D‐IOD, three participants each performed 9.7 days of 7 h of paid work despite complaints: A total of 67.9 h. Since they were still able to work at 60%, the 40% productivity loss sums to 27.16 h, which amounts to €991.88 for each of these three patients considering a productivity costs of €36.52 per hour [28].

During the period that the C‐IOD was worn two participants performed each 15 days of 7 h of paid work despite complaints. Since they were able to work at 90%, the 10% productivity loss amounted to 10.5 h each, or €383.46, for each of the two participants.

##### 3.4.4.3 Productivity Loss in Case of Unpaid Work

Related to unpaid work, nine individuals were able to perform less unpaid work for a period of 16.6 days while wearing a 3D‐IOD. When wearing the C‐IOD, seven persons were hindered for 26.7 days.

Hours that had to be taken over in terms of housekeeping or informal care were 1.6 h for each of the nine 3D‐IOD wearers (in total: €23.54) and 2.8 h for each of the seven patients wearing a C‐IOD (in total: €41.19). To value the costs of unpaid work, the costs of housekeeping (€14.71 per hour) were used, as advised by the National healthcare institute in the Netherlands [28].

##### 3.4.4.4 GHRQoL: EQ‐5D‐5L

The ANOVA analysis of the EQ‐5D‐5L showed evidence for “intersubject variability” (*p* < 0.05), but not for “treatment” (*p* = 0.856) nor “center” (*p* = 0.099, “period” (*p* = 0.818) or “carryover” effects (Table 6). So, utilities were comparable between C‐IOD and 3D‐IOD confirming part of our main hypothesis (similar effects on EQ‐5D‐5L). The paired *t*‐test elucidated no differences in general GHRQoL between C‐IOD (*M* = 0.840, SD = 0.177) and 3D‐IOD (*M* = 0.837, SD = 0.156) (*N* = 31): *p* = 0.880).

**TABLE 6:** ANOVA analysis of the EQ‐5D‐5L for the effects of center (Nijmegen vs. Breda), sequence (start with C‐IOD vs. 3D‐IOD), period (first vs. second period), treatment (C‐IOD vs. 3D‐IOD) and carryover effects.

	Number of objects = 63		R‐squared = 0.055		
	Root MSE = 0.224		Adj R‐squared = −0.011		
Source	Partial SS	df	MS	F	Prob > F
Model	0.167	4	0.042	0.84	0.507
Center	0.140	1	0.140	2.81	0.099
Sequence	0.003	1	0.003	0.06	0.800
Period	0.003	1	0.003	0.05	0.818
Treatment	0.002	1	0.002	0.03	0.856
Carryover	0	0			
Residual	2.900	58	0.050		
Total	3.07	62	0.049		

Abbreviations: df = degrees of freedom, F = F‐ratio, MS = Mean Square, SS = sum of squares.

**GRAPH 1:**
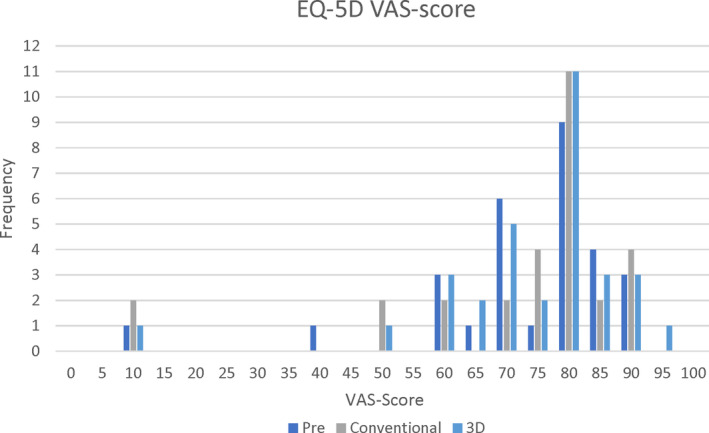
Frequency of general Health VAS‐score.

The mean VAS‐scores for general health when wearing a CD (at baseline) and after wearing the C‐IOD and 3D‐IOD measured 73.79, 71.97, and 74.59, respectively (Graph 1). These differences were not significant: Baseline versus C‐IOD, *p* = 0.735; baseline versus 3D‐IOD, *p* = 0.855; C‐IOD versus 3D‐IOD, *p* = 0.590.

Both while wearing the C‐IOD as well as wearing the 3D‐IOD, one patient scored 10 points or lower for general health on the VAS (1–100). The median, however, was 80 points for both overdentures as well as at baseline.

##### 3.4.4.5 GHRQoL: SF‐6D

**GRAPH 2:**
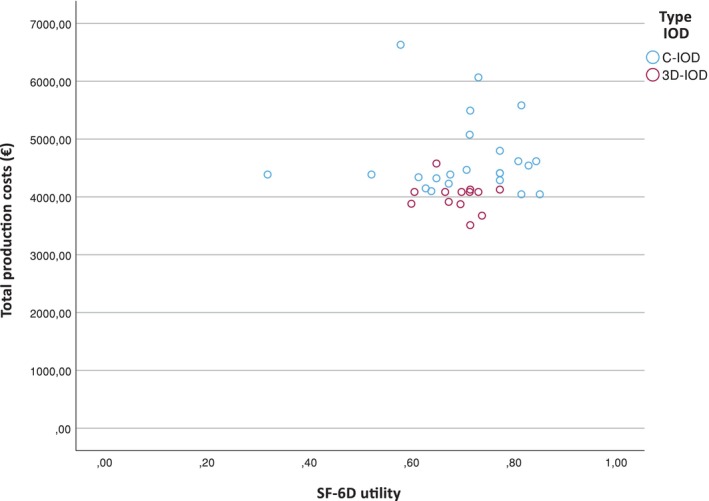
Distribution of costs and SF‐6D utility.

The SF‐6D (Graph 2) yielded equal utilities for the C‐IOD and 3D‐IOD (0.706 vs. 0.711). However, because the mean total production costs were significantly higher for the C‐IOD than for the 3D‐IOD (€4700.33 vs. €4030.61 (< 0.001)), the 3D‐IOD was considered as cost saving and therefore the most efficient modality.

### 3.5 Budget Impact Analysis (BIA)

Data provided by the National Health Care Institute indicates how often certain treatment codes were reimbursed (Table 7). There was a specific code for “solitary lower IOD” and “solitary upper IOD,” as also the combination of “upper CD + lower IOD‐2,” meaning that in the upper jaw a full CD was placed and at the same time an IOD‐2 in the lower jaw. This treatment method is often executed, because when edentulous patients complain about the retention only of their upper CD, the Dutch guideline recommends first making an IOD‐2 in the lower jaw [29].

**TABLE 7:** Numbers and costs of IODs as reimbursed by the Dutch assurance companies between 2013 and 2021.

Codes	Description	2013	2014	2015	2016	2017	2018	2019	2020	2021
Upper CD + lower IOD‐2	Numbers x 1000	80.1	90.7	100.1	105.2	95.4	107.1	110.0	93.9	106.1
Solitary lower IOD	Numbers x 1000	13.6	14.3	15.7	17.0	17.3	21.0	21.6	21.2	23.6
Solitary upper IOD	Numbers x 1000	11.1	12.4	14.0	14.9	16.2	20.3	21.3	21.3	23.9

So, in total 129.700 (106.100 + 23.600) mandibular IODs‐2 were placed in 2021. For the edentulous maxilla, 23.900 IODs were constructed, meaning IODs‐4 or IODs‐6 in 2021 (Table 7).

In 2021, maximum reimbursement for a C‐IOD‐2, C‐IOD‐4, C‐IOD‐6 was €1458, €2625, and €3375 [27]. However, when digital manufactured 3D‐IODs would have been used, these costs could be reduced to €1185, €1541, and €1798.50, respectively.

After consultation with experts in the field of implantology, it was estimated that for the upper jaw in ⅔ of the cases an IOD‐4, and in ⅓ of the cases an IOD‐6 was made, and that ⅔ of the practitioners used bar‐retained, and ⅓ single abutment constructions. In the lower jaw, it was supposed that in 80% of the cases (*n* = 103.760) a bar was used, and in 20% (*n* = 25.940) single attachments.

Digital workflow reduced costs for the upper IODs with €19.907.613 and for the lower IODs with €28.378.360, together €48.285.973 (Table 8).

**TABLE 8:** Numbers and lab costs of C‐IODs which were reimbursed by the assurance companies in the Netherlands for 2021. Costs and savings were specified for the upper IOD‐4 and IOD‐6, as well as for the mandibular IOD‐2. Total costs point to possible savings on a yearly base: 25%, 50%, 75%, or 100% (scenarios 1–4).

Maxilla	Estimated number of IOD placements	Lab costs C‐IOD	Lab costs 3D‐IOD	Total lab costs C‐IOD	Total lab costs 3D‐IOD	Savings 2021
IOD‐4 on bar	10.622 (⅔ × ⅔ × 23 900)	€2625	€1541.5	€27.883.005	€16.373.813	€11.509.242
IOD‐6 on bar	5.311 (⅔ × ⅓ × 23 900)	€3375	€1798.5	€17.924.821	€9.551.834	€8.372.987
Mandible	Estimated number of IOD placements	Lab costs C‐IOD	Lab costs 3D‐IOD	Total lab costs C‐IOD	Total lab costs 3D‐IOD	Savings 2021
IOD‐2 on bar	103.760 (0.8 x 129 700)	€1458	€1184.5	€151.282.080	€122.903.720	€28.378.360
						Accumulated savings 2021
Scenario 1				**100% savings**		**€48.260.589**
Scenario 2				**75% savings**	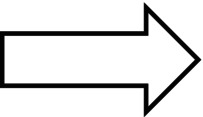	**€36.195.442**
Scenario 3				**50% savings**		**€24.130.295**
Scenario 4				**25% savings**		**€12.065.147**

In total four scenarios were presented. Since a digital workflow takes time to implement in the daily clinic, it was estimated that in the next year 25% of treating dentists will switch to a digital workflow, and so on.

## 4 Discussion

### 4.1 Cost Consequence Analysis (CCA)

#### 4.1.1 Costs Made Within Healthcare—Initial Costs for Mandibular IODs

Costs for a 3D‐IOD were 20% lower than for a mandibular C‐IOD (€1695.03 vs. €2106.35: *p* < 0.001).

Various economic evaluations to mandibular IODs‐2 underlined their cost effectiveness, meaning that the gain in OHRQoL outweighs the costs [30, 31, 32, 33, 34, 35]. These evaluations comprised also the implant treatment, so no comparison could be made on level of only the prosthetic part of the IOD, the attachment system Although absolute costs for the IOD (implants included) differ, it was claimed that the ratios among the various solutions are consistent in literature: Compared to a CD, an IOD‐2 is about 2.4 times more expensive, and an IOD‐4 around 6 times [33, 36]. When adding the costs for implant treatment in our study, prices for a CD, C‐IOD‐2, and C‐IOD‐4 were €1263.54, €3378.74, €5535.72 showing a ratio of 1:2.7:4.4 [27]. When incorporating digital techniques, 3D‐IOD‐prices including implants become even more favorable: A ratio of 1: 2.5: 3.5 can be calculated for a CD (€1263.54) versus 3D‐IOD‐2 (€3121.47) and 3D‐IOD‐4 (€4452.22).

#### 4.1.2 Costs Made Within Healthcare—Initial Costs for Maxillary IODs

For the maxillary bar‐retained C‐IOD and the bar‐retained 3D‐IOD total costs were €3659.70 and €2239.41, including laboratorial costs of €2846.60 and €1644.30 respectively.

Few articles have been published about the costs of maxillary IODs. Listl et al. (2014) assessed the cost‐effectiveness of a bar‐retained maxillary IOD‐4 versus a bar‐retained maxillary IOD‐6: In Germany (2014) material and lab costs, thus without implants, were €4507.82 for the maxillary IOD‐4, and €5070.30 for the maxillary IOD‐6 [37]. In contrast, in our study material and lab cost were nearly three times cheaper, namely €1541.50 for the 3D‐IOD‐4 and 1798.50 for the bar‐retained 3D‐IOD‐6.

With respect to cost effectiveness analyses (CEA) in relation to maxillary IODs, in a recent systematic review, Ghiasi et al. incorporated only two articles, both from Zitzmann et al. [31, 32, 38] They concluded that based on these two publications, which evaluated a single patient cohort, no conclusions could be drawn [38].

Our results showed that the price for a maxillary C‐IOD‐6 on Locators was lower than for a bar‐retained C‐IOD‐6, which can be explained by the fact that the Locator system is an off‐the‐shelf product, while the bar‐retained construction is individually fabricated. However, since a bar‐retained 3D‐IOD‐6 was less expensive, its price became close to a C‐IOD‐6 on Locators.

Regarding IOD‐attachment systems, Sutariya et al. (2021) concluded that bar‐attachment provided the most superior retention, together with a minor prosthetic follow‐up [39]. Also, Ciftci et al. (2023) advocated the screw‐retained bar [40]. Only in case of limited inter arch space and on condition of parallel implant placement, the move to the Locator system can be made [41].

#### 4.1.3 Costs Made Within Healthcare—Maintenance and Repair

It should be recognized that the replacement of a CD with an IOD do increase both the objective and subjective chewing capacity [42]. For an IOD‐4, for example, bite forces (334 N) went four times up compared to a mandibular CD (76 N) [43].

With increased bite forces, the weakest link is always at risk; in our study this turned out to be the mandibular IOD‐2 for which the most complications were registered. The bar attachment in the lower C‐IOD was particularly susceptible to damage, namely loosening or loss of the ball attachment itself, or the acrylic VKS attachment. For the 3D‐IOD‐2, loose screws (11x) and even screw fractures (7x) were seen. All these negative events occurred only in five patients, all of whom were diagnosed with bruxism. Screw fracture is a serious complication, especially if the screw cannot be removed properly.

Some authors reported that mechanical complications had no impact on the satisfaction and quality of life of patients treated with complete arch implant‐supported prostheses, however, these costs should also be implemented in a cost analysis [44].

Ghiasi et al. (2022) stated that, due to the relative low maintenance and repair costs, large differences in initial cost remain the main component in the total costs, even over time [4]. This was corroborated in our study: Initial production costs outweighed the maintenance and repair costs substantially. In contrast, major complications in need of extra interventions, such as implant installation due to implant loss, or the fabrication of a new IOD, could indeed affect long‐term costs [45]. In this perspective follow‐up longer than 1 year is relevant.

#### 4.1.4 Costs on Patient Level Made by Patients/Family, Real Travel Costs, Out‐Of‐Pocket Expenses, and Time Costs

Making a C‐IOD in both upper and lower jaw took 19.2 h per patient, both in treatment and travel time, reparations included. A 3D‐IOD can be made in two third of that time (12.3 h).

Only eight (25%) participants had a paid job. Productivity loss caused by treatment sessions and travel time were substantially lower for the making of a 3D‐IOD: €383.47 versus €991.89 with a C‐IOD.

Productivity loss of informal caregivers, for example, the patients' children accompanying or driving their parents to the appointments, was not applicable in this study since every patient was still able to attend the dentist appointment on their own. Especially in an aging population however, it can be beneficial to bear in mind this aspect of microcosting as well.

#### 4.1.5 In Other Sectors Than Healthcare: Costs of Productivity Loss due to IOD‐Manufacturing While Working (Paid and Unpaid)

During the research period of 2 years, also the average time of productivity loss per patient due to illness was relatively low. For the 3D‐IOD: €642.75 in case of three persons and €383.47 for the C‐IOD in case of 2 persons.

To our knowledge there are two other studies on productivity loss in relation to dental treatment. Since one focused on absenteeism in relation to dental checkups [46] and the other encountered practical limitations in specifying the oral conditions related to the experienced time loss [47], their data could not be related to our research.

##### 4.1.5.1 EQ‐5D‐5L and SF‐6D

To measure the health benefits often the EQ‐5D‐5L questionnaire is used. As shown in our study, this method did not score any changes between groups. In contrast to the sufficient validity in case of persistent orofacial pain [48], the EQ‐5D‐5L apparently appears to be too insensitive to recognize oral health changes, which underlines the importance of oral health disease specific questionnaires [49]. If these were also preference‐based, this could furthermore improve economic evaluations in the field of OHRQoL.

The effect of IOD‐type was thought to be limited to the exact period that one of the IOD‐types was worn. Therefore, as carryover effects were not anticipated, no washout period was integrated [50].

##### 4.1.5.2 Patients

The reasons why patients want their CDs changed to IODs on the one hand, and the willingness and ability to undergo surgery and pay the costs on the other hand, determine the final choice between the different types of rehabilitation. In this decision‐making process, patient's socio‐economic status and geographic location play a pivotal role as do the costs for implant treatment, as it differs substantially between countries and healthcare systems [33, 51]. Still today, essential information about and cost‐effectiveness of different treatment options for edentulous situations lacks [52].

##### 4.1.5.3 Implants and Prosthetics

Maxillary implant SR was 97.2% after 2 years of functioning, the mandibular SR 100%, also the prosthetic SR was 100% for both upper and lower IOD, which corroborates the findings in a recent review reporting an SR rate of 98.3% for six splinted implants supporting an upper IOD combined with a prosthetic SR of 97.9% [53]. Also, Milisavljevic et al. reported comparable high SR for both maxillary and mandibular IODs [54].

The decision to choose for a bar‐retained IOD, or an IOD on single abutments rests with the dentist. The reason to choose a bar in the upper jaw is twofold: Bars that splint the implants, induce a higher implant SR than an IOD on single abutments [55]. Furthermore, nonparallel implants only can be corrected using bars [41].

The argument, that Locators are better cleanable, has recently been contradicted. In a 5‐year follow‐up study of maxillary IODs, the peri‐implantitis incidence was 25.8% in the solitary attachment group and only 5.1% in the bar‐retained group. Furthermore, it was concluded that for maxillary IODs‐4, fewer implants survived in the case of solitary attachment (89.5%, *p* = 0.027) than in the case of bar‐retained attachment (96.3%). In addition, the IOD‐SR was 95.0% for bar‐retained and 91.3% for solitary attachment [56].

#### 4.1.6 Budget Impact Analysis (BIA)

To provide more patients access to the benefits of IODs, costs need to decline, or public funding needs to increase. Key in lowering costs is innovation of both treatment protocols and manufacturing (CAD/CAM) techniques [14].

The application of digital techniques significantly reduced the costs of a C‐IOD. It can be highlighted that a high quality (bar‐) attachment system can be delivered at the same price as a simple (Locator‐) attachment system when using digital techniques.

To our knowledge, this is the first BIA performed in the field of oral implantology and prosthetics. In dentistry, only one other BIA was found, comparing the effect of fissure sealants with the use of fluoride varnish in children at high caries risk [57]. Also is the field of oral maxillofacial surgery (OFMS) a recent BIA has been published, comparing orthodontic protraction versus orthognathic surgery to advance the maxilla in patients with cleft lip and palate for the treatment of Class III malocclusion, concluding that orthodontic protraction was the less expensive and less invasive treatment modality [58]. BIAs are widely accepted within other medical disciplines, such as cardiology in the treatment of heart failure [59].

Based on our BIA, it was concluded that in the Netherlands about 50 million euro can be saved on an annual basis, thereby enabling insurance parties to provide more patients with an IOD for the same budget. Furthermore, 3D‐IODs can be made in less sessions and are, therefore, more affordable for a patient.

## 5 Conclusion

Implementing a 3D workflow in the production of IOD's supplies patients with a high‐quality 3D‐IOD at lower costs. The introduction of an oral health related preference‐based measure would improve economic evaluations in the field of OHRQoL.

## Author Contributions

T.V.W. conceptualized the project idea, conducted the literature search, and drafted the manuscript. F.D. designed the digital workflow and gave technical advice. B.O. advised on the clinical design of the study. E.A. controlled the CCA statistically and gave technical advice. E.A., T.M. and G.M. critically reviewed and revised the manuscript.

## Conflicts of Interest

The authors declare no conflicts of interest.

## Data Availability

The data that support the findings of this study are openly available in DANS at https://doi.org/10.17026/dans‐25s‐6cdk, Persistent identifier 10.17026/dans‐25s‐6cdk.
